# Adoption of a Serious Game in the Developing of Emotional Intelligence Skills

**DOI:** 10.3390/ejihpe10010004

**Published:** 2019-07-20

**Authors:** Fernando Almeida

**Affiliations:** Faculty of Engineering, University of Porto, INESC TEC & ISPGAYA, 4200-465 Porto, Portugal; falmeida@ispgaya.pt or

**Keywords:** emotional intelligence, serious game, FLIGBY, flow, game-based learning, academic assessment, mixed methods

## Abstract

Emotional intelligence is intrinsically associated with the ability to understand, manage, and express feelings and deal with other people’s emotions. This competence is essential for the formation, development, and maintenance of personal and professional relationships. Furthermore, emotional intelligence can be extensively worked out and developed over time, which allows each individual to become a better professional. Nevertheless, the perception that higher education students have about the importance of emotional intelligence remains residual and there are few contexts that allow them to develop emotional intelligence skills. In this sense, this study proposes the use of a serious game to assess and develop emotional intelligence skills in the context of an entrepreneurship discipline attended by multidisciplinary students from the courses of management and computer engineering. The performance of students is measured and discussed considering a mixed methods approach. The findings indicate the existence of a correlation between the player’s emotional intelligence skills and his performance in the game, and this occurrence is common to students regardless of their course, gender, age, and number of years of professional experience. The study also explores the importance of emotional intelligence considering the distinct profile of students.

## 1. Introduction

Games are an integral part of the civilization and have always been a very popular form of entertainment among the public of all ages, although they are more related to children and young people. In different cultures, games are also an essential activity used as a teaching strategy [1]. Currently, many games are developed precisely with the purpose of helping the teaching of some specific theme, that is, learning is the focus on the development of concepts, skills, techniques, etc. [2,3,4]. In this case, the game becomes serious by including these elements.

For the authors in Reference [5], serious games are games that do not present entertainment as a primary goal. Reference [6] states serious games should offer scientific and social knowledge to students and professionals, thereby improving the skills and techniques through virtual activities. Serious games have the purpose of learning and changing behavior, and should be based on three elements: purpose, content, and design [7]. Serious games are highly interactive and motivating products. When playing a game, a series of events is generated from the delineation of a narrative and trigger emotions, pleasures and unique challenges, for the exploration of this narrative. The intrinsic motivation for the use of serious games depends on multiple factors [8,9,10]: (i) autonomy and control; (ii) immediate feedback; (iii) learn from the mistakes; (iv) collaboration and/or competition between players; (v) flexibility of the proposed challenges; and (vi) increased motivation through the challenges posed to the player.

Game-Based Learning (GBL) is a pedagogical methodology that focuses on the design, development, and application of games in education. GBL is part of the general concept of serious games and has been used successfully in several areas such as health, planning, or management [11,12,13]. In this sense, it is important to explore the adoption of serious games in new areas that have been minimally explored, namely in the development of emotional intelligence (EI) skills among higher education students. EI is defined as the ability to identify and manage one’s own emotions and those of others [14]. With regard to the study in Reference [15], EI is more important than cognitive intelligence, and is one of the key factors for personal and professional success. Therefore, the aim of this study was twofold: (i) to explore the association level between the performance of the students in the emotional intelligence dimension and their final academic performance in the discipline of entrepreneurship; and (ii) to assess the relevance of contextual variables (i.e., course, gender, age, and years of professional experience) in the students’ performance in the “emotional intelligence” component. The manuscript is organized as follows: initially, a theoretical contextualization on the relevance of EI and on processes to assess and evaluate the player’s performance in serious games is given. The materials and methods used in this study are then presented, followed by the results and discussion on their relevance to the scientific community. Finally, the main conclusions of the study are listed and some items for future work are suggested.

### 1.1. The Relevance of Emotional Intelligence

The concept of EI aggregates two fundamental ideas: that emotions can make thought more intelligent and that a person can think intelligently about emotions [16]. EI is understood as a set of skills related to the perception, expression, and regulation of emotions in oneself and in others, as well as the use of these skills to motivate, plan, and achieve goals in life [16]. In the study conducted in Reference [16], the EI is explained by means of a system of four organized components: (i) perception, evaluation, and expression of emotion; (ii) emotion as a thought facilitator; (iii) understanding and analysis of emotions and use of emotional knowledge; and (iv) reflective control of emotions to promote emotional and intellectual growth. Exploring these dimensions facilitate the understanding that EI is both interpersonal and intrapersonal. Interpersonal, when referring to interactions between individuals, perceive emotions in others, manage the emotions of others in social exchanges. Interpersonal, when referring to the individual himself, the way in which he/she recognizes and processes emotional information, and how this affects his/her thoughts and behaviors.

Goleman’s theory on EI reveals that emotions influence people’s lives and can contribute to good interpersonal relationships [14]. The book in [14], considers that emotionally balanced people have more opportunities to become leaders, than the individuals who present high values of Intelligence Quotient (IQ). In this sense, it is possible to infer that IQ alone is not enough for success. In fact, Goleman’s study suggests that almost 90% of the skills needed for professional success are emotional and social [14]. Goleman, in his research on 200 multinational companies, found that effective leaders are destined for a high degree of EI. Therefore, without EI, managers can have excellent training, an incisive mind, and an inexhaustible source of good ideas, but they will not become a great leader [17].

Another well-known model was proposed by Bar-On, in which EI is seen as a composed model. Bar-On’s non-cognitive model looks at emotional intelligence as a set of social skills, non-cognitive skills, and competencies that influence the ability to be successful in dealing with environmental demands and pressures [18]. According to this model, EI is a set of non-cognitive abilities, knowledge, interrelated emotional and social skills that determine how effectively individuals understand and express themselves, as well as understand others and relate to them, and deal with the demands and pressure of daily life to be successful [18].

Furthermore, the measurement instruments for evaluating EI that have been developed in the market are subject to discussion among researchers. There are several measures of EI that allow exploring the psychometric characteristics of individuals. The content of EI tests varies according to the different theoretical interpretations and conceptualizations. In [19], these tests are divided into two groups: those that derive from self-portraits of daily behaviors (trait emotional intelligence) and those that depend on objective performance in controlled experimental situations (ability emotional intelligence). The former asks the individual to report on his own emotions caused by different situations and, therefore, the individual progressively tests his level of EI. The latter asks the individual to solve tasks related to the recognition of their own emotions and other people’s, and to identify socially appropriate responses. The performance measures enable an external evaluation of the performance and minimize the potential occurrences of biased responses [20]. In the performance measures, the construction criteria are identical to the capacity tests, while in the self-report model, the construction criteria are similar to those of the personality tests [19].

### 1.2. Performance Assessment in Serious Games

The process of collecting data, measuring, and evaluating performance is often neglected in serious games. In [21], this situation can affect the motivation of the learning process and consequently the efficiency of the game. In this sense, it is important that serious games include objective and broad criteria on the process of measuring the player’s performance. Furthermore, immediate feedback is another essential element for the player to feel in control of the game. The study in Reference [22] emphasizes the necessity that serious games provide immediate feedback throughout the training and in the end, to reinforce the successes and correct the errors. The model proposed in [23] includes both evaluation criteria and feedback. The evaluation criteria should include: (i) several types of performance evaluation (e.g., diagnostic, formative, self-evaluation); (ii) evaluation tools based on well-defined objectives; (iii) continuous measurements and evaluations of the process; (iv) evaluation of the training program; and (v) the dimensions of human errors. On the other side, feedback criteria should include: (i) immediate feedback throughout the training and in the end; (ii) reinforce extrinsic motivation with verbal or tangible rewards (e.g., scoring, positive feedback); (iii) attribute successes and failures to the player; (iv) reinforce the player’s success in completing a task; and (v) unexpected rewards in order to foster player engagement.

Serious games can include multiple assessment elements. The study in [24] demonstrates that the process of evaluating a player’s performance can include two perspectives: (i) the pedagogical aims (i.e., formative, and summative) and the implementation site (i.e., inside, and externally). Formative assessment is carried out throughout the process, in all learning situations, on each objective. Therefore, the formative assessment identifies situations of poorly achieved learning and informs about corrective measures to be taken. Summative assessment makes it possible to carry out an analysis of the competencies acquired by the students at the end of the training. In addition, one of the objectives of summative assessment is the serialization of students and, therefore, there is a classification of students’ performance. Whatever the scales, the classification should be made explicit to the students and the reasons for choosing a particular scale should be discussed. Summative assessment proves to be a relevant instrument in decision making, and allows for the comparison of the overall results of training programs applied to similar or different groups of learners [25,26].

The assessment of the player’s performance can be done after the player has completed the game or embedded in the game itself. Assessment after learning in a GBL environment is focused on the identification and evaluation of outcomes. This approach is typically built through questionnaires or structured or semi-structured interviews. One of the advantages of this approach is to enable the comparison of the player’s individual learning outcome to other players or experts [27]. However, this approach does not allow drawing conclusions on the cause of a possible incorrect result and does not allow obtaining instant feedback [27]. Another alternative is to define a process of evaluation of the player’s performance while he/she is playing. Several benefits are pointed out in this approach namely: (i) detailed insights into underlying learning processes; (ii) allows tracking motivational, emotional, and meta-cognitive characteristics of the player; and (iii) gives immediate feedback on the player’s actions [27,28]. Finally, this approach also allows building an adaptive game environment according to the actions and difficulties experienced by the player [29,30]. In this situation, the game would be customized according to the actions taken by the player and the player’s learning would necessarily be individualized.

## 2. Materials and Methods

### 2.1. Instruments

The FLIGBY serious game was used in the process of developing and assessing ability emotional intelligence skills. FLIGBY is based on the Flow theory, a core concept in positive psychology. FLIGBY allows players through an immersive and dynamic environment to apply the theoretical concepts of Flow into daily management practices [31]. One of the fundamental objectives of this game is to create a business environment conducive to teamwork, in which all employees feel involved and committed to the mission of the company.

The concept of Flow theory was proposed by Professor Mihaly Csikszentmihalyi [32]. In his research, it became clear that there are common elements that allow people to achieve a state of absolute focus even if they have different professions, characteristics, and cultures. In a state of Flow, there is total involvement of the person in what he/she does, a sense of serenity and motivation that allows him to work without having a sense of losing time [32]. The main conditions that favor the state of Flow are: (i) a work environment is to have clear and defined objectives, (ii) established rules, (iii) challenges according to the person’s skills, as well as (iv) feedback and encouragement on the performed work [32]. Therefore, understanding Flow can make a positive contribution to the organizational environment, since this condition promotes better performance and produces commitment and motivation both individually and in teams.

According to [33,34], Flow contributes to: (i) increase the individual’s emotional, cognitive and social capacity; (ii) increase employee performance; and (iii) increase motivation, sense of engagement, and perception of personal growth. Results from the study in [35] allow us to identify that there is a strong correlation between Flow and the development of competencies. In fact, when an individual is in the state of Flow, he or she will also be working to develop excellence in the activity he or she is performing. However, for an individual to be in the Flow state, the degree of challenge offered by the activity must be compatible with the person’s current level of competence. Consequently, when the competence increases, the challenge will also need to increase in order for Flow to continue to be generated. This situation allows the creation of a virtuous bond in which the challenge and competence gradually increase.

At FLIGBY, the player has the double challenge of managing a company in the wine area and achieving a state of harmony and collaboration among the company’s employees. FLIGBY is composed of 21 scenarios, which take approximately 7–9 h to complete. Throughout the game, the player makes approximately 150 decisions that affect the performance and motivation of each employee. In fact, there is gradual learning of the payer along the game, and in the fourth scenario, the player has the possibility to return to the beginning of the game. Approximately 75% of the players took this decision, which means that there was a perception that some wrong decisions were made in the early scenarios. Furthermore, MR. FLIGBY’s role is fundamental in the game. He assumes the role of virtual coaching and gives feedback on the player’s performance. Achieving the state of Flow, whether individual or collective, is challenging as players have very different personalities. In fact, the state of Flow can be achieved in two ways: by making decisions that affect colleagues so that each of them individually can achieve the state of Flow; or by adjusting decisions and promoting a favorable corporate atmosphere for the team’s Flow.

The interface of FLIGBY is very appealing and interactive, in which the player interacts with the various characters of the game. The player has to make several decisions over the 23 scenes offered by the game. After the completion of each level, the Mr. FLIGBY character, who assumes the position of coaching, gives feedback on the actions of the player in the game. This feedback is relevant for the player to reflect on the decisions made, seeking with this that the player goes progressively learning throughout the game.

### 2.2. Sample

The FLIGBY serious game was adopted in the context of the entrepreneurship discipline. This discipline is attended by multidisciplinary students from the undergraduate courses of Management and Computer Engineering. In order to allow the attendance of all students, particularly those that are working, two classes were created: one during the 14:30–17:30 slot and the other during the 20:00–23:00 slot. The discipline of entrepreneurship presents an ideal scenario for the use of FLIGBY, because from the use of this game students can test various scenarios of management of an organization. Furthermore, one of the determining factors for the success of the entrepreneur is the development of their EI [36,37]. Therefore, as in other areas of business activity, emotional competence is a relevant factor to define between the success and failure of an entrepreneur. According to [38], one of the first steps to conquer EI is to go through a process of self-knowledge that will help the individual to better understand their strengths and weaknesses. In fact, admitting one’s own limitations is a sign of maturity in any relationship, especially in the professional environment. The use of FLIGBY will allow students to recognize their own skills and limitations and, through it, deal more easily with their own emotions, eliminating negative reactions and seeking new forms of interaction with people.

**Figure 1:**
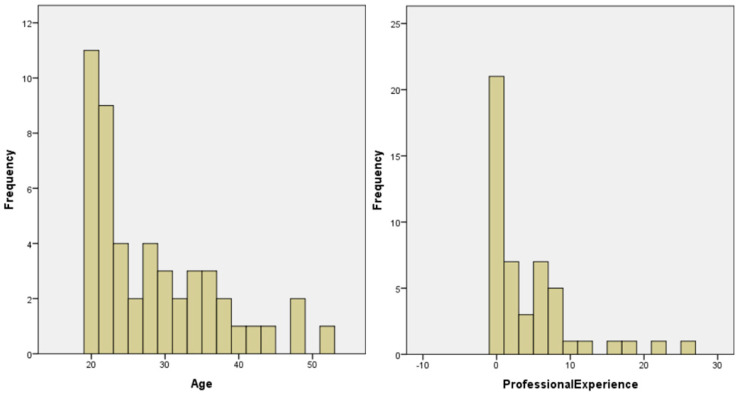
Histogram of the relative frequencies of participant demographics.

A total of 51 students were initially expected in the sample. However, two did not complete the game by dropping out. In this sense, the considered sample included data from 49 students. Table 1 presents descriptive statistics of the sample’s elements, considering the course, gender, age, and years of professional experience of students. The majority of the students came from the computer engineering course and are males. This situation is mainly accentuated by the existence of only four females in the computer engineering course. More than 75% of the students are under 35 years of age. Although ~43% of the students had no work experience, there was a wide dispersion of years of experience. This situation is evidenced in the histogram shown in Figure 1. The students’ professional experience focuses mainly on secretarial, logistics, programming, and systems administration. It should be noted that the majority of the students who attended the entrepreneurship discipline were simultaneously working in the labor market.

### 2.3. Research Design

In the development of this study, mixed methods were adopted through a triangulation design approach. According to [39,40], this approach allows the simultaneous use of quantitative and qualitative methods in the exploration of distinct but complementary information on the same topic. For this purpose, the quantitative data were collected in the first instance, and the collected qualitative data served to complement the information previously compiled.

The quantitative data were obtained using the Master Analytics Profiler (MAP) provided by FLIGBY. Player performance is measured considering 29 key performance indicators (e.g., active listening, analytical skills, business-oriented thinking, feedback, emotional intelligence, etc.). Precisely one of the measured indicators is the “emotional intelligence” dimension, that in the game is assessed considering the player’s ability and readiness to perceive, express, and regulate emotions in oneself and in others. Each one of the eight characters of the game has distinct functions in the company and personalities that are often conflicting and incompatible. Table 2 presents a summary of the functions of each of these characters.

Qualitative data were obtained through a semi-structured group interview that was conducted after the game’s conclusion. This interview was audio-recorded and took place in the classroom and lasted approximately 60 min. All participant students gave their informed consent for inclusion before they participated in the study. Only two students that played FLIGBY did not participate in the interview group due to illness. This approach allowed greater interaction between all students and the teacher, rather than the alternative of using individual interviews. At the time of the semi-structured interview, the students already had information about their final assessment in the game considering the 29 key performance indicators provided by FLIGBY, which also includes assessment in the “emotional intelligence” dimension. The semi-structured interview is characterized by the existence of a previously prepared script that serves as a guideline for the development of the interview [41]. According to [42], this approach allows the in depth exploration of a theme and introducing new issues according to the comments and feedback collected by the interviewees. In [42], this approach is especially recommended for interviews with groups, and therefore its adoption is adjustable in the context of a classroom in which the students were present to attend the discipline of entrepreneurship. Table 3 presents the structure of the semi-structured interview. Three dimensions were considered: (i) background; (ii) assessment; and (iii) forecast. The background dimension seeks to understand the students’ current knowledge about EI; the assessment dimension analyzes the students’ perception of their performance in the FLIGBY game; and the forecast dimension intends to explore the impact that emotional skills may have on students’ careers.

## 3. Results

### 3.1. Quantitative Analysis

The IBM SPSS Statistics V.21 was used in the statistical analysis of FLIGBY’s prospective quantitative data. The IBM SPSS software allows statistical analysis of data considering several quantitative methods of descriptive analysis, regression, and inferential statistics. First, a reliability analysis considering the data obtained from the MAP was performed (Table 4). For that, four reliability functions were considered: (i) Cronbach’s Alpha; (ii) Cronbach’s Alpha Based on Standardized Items; (iii) Spearman-Brown Coefficient; and (iv) Guttman Split-Half Coefficient. Stapleton et al. [43] considered it essential to evaluate the lower limit of the internal consistency of a group of variables or items, and this value must be higher than 0.7 to determine accurately which variables are included in the internal consistency analysis.

**Figure 2:**
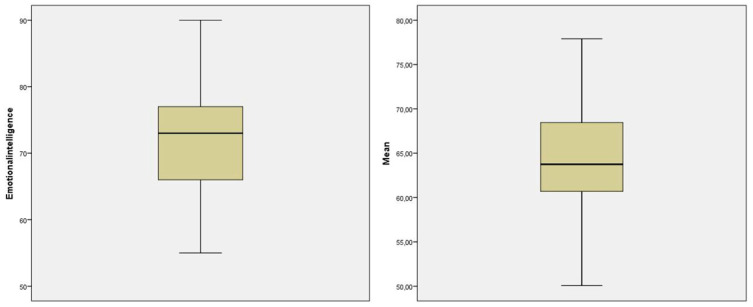
Boxplot diagram.

Table 5 provides an analysis of the sample data considering several statistical indicators such as mean, median, mode, standard deviation, skewness, kurtosis, percentiles, and range. The results indicate that the sample has negative asymmetry, i.e., there is a higher concentration of values in the upper part of the sample (Figure 2). Comparing this result with the average performance of students in all 29 key performance indicators of FLIGBY, some relevant oscillations can be observed: (i) the asymmetry of the behavior of the two indicators is opposite; (ii) the students’ performance in EI dimension is higher than the average of the other dimensions; and (iii) there is a greater dispersion and range of results in the EI dimension. It is also pertinent to explore whether the students’ performance in the game was different from the benchmark that includes data from undergraduate and post-graduate students from several worldwide institutions. The performance of the students in the emotional intelligence dimension aligned with the benchmark (mean = 72). In contrast, the performance of students in this sample (mean = 63.99), considering the other dimensions, was lower when compared to the benchmark (mean = 67).

A linear regression analysis was also performed between the independent variable “emotional intelligence” and the dependent variable “students’ final score” considering all the dimensions of MAP. Table 6 shows the R value, R Square, Adjusted R Square, and Std. Error of the Estimate. There is a significant correlation between the two variables under study (i.e., R = 0.808), in which 65.3% of the variance is explained by the model. However, there is 34.7% of the regression variance that does not depend on the variables under study. In addition, the *p*-value was calculated to explore whether there is a significant difference between the mean of both groups (i.e., emotional intelligence dimension, and all dimensions). A significance level of 0.05 was adopted. The *p*-value of the sample is below 1 × 10^−3^, which indicates that there were significant differences in the mean between the two groups. This result indicates that the performance of students in emotional intelligence dimension is a determining factor in the final assessment of students in the game. In this context, we can also infer that the students who perform best in emotional intelligence dimension are also those who perform best in other MAP dimensions.

Finally, an ANOVA analysis was performed to assess the importance of the four dimensions identified in Table 1 (i.e., course, gender, age, and years of professional experience) in the students’ performance in the “emotional intelligence” component. For this purpose, a significance level of 0.05 was adopted. The null hypothesis states that all population means (factor level averages) are equal, while the alternative hypothesis states that at least one is different. The results in Table 7 indicate that the variance of the sample elements is homogeneous and none of the considered dimensions is statistically relevant in the behavior of students’ performance in the “emotional intelligence” component.

### 3.2. Qualitative Analysis

One of the most widespread methods for analyzing the outcomes of a semi-structured interview is by conducting a thematic analysis. There are several forms of structuring and synthesizing qualitative data, and in this study we adopted the model proposed by [44], in which the process is decomposed into six phases: (i) familiarizing the researcher with the background theory; (ii) generating initial codes; (iii) search for themes considering the various codes generated in the previous step; (iv) revision of the themes and refinement of the model; (v) final definition of the themes; and (vi) writing the report. Table 8 shows a correspondence between the dimensions established in Table 3 and the results obtained from the thematic analysis process.

## 4. Discussion

EI proved to be a topic still little known by students, especially by the students without professional experience. Two misconceptions emerged associated with this theme: (i) on the one hand, EI was perceived as an innate ability that did not require development; and (ii) on the other hand, EI was perceived as a concept with little practical applicability. This incorrect perception was higher among students without professional experience and common to both courses.

Overall, students experienced some difficulties in identifying the advantages of EI in their academic life. The interaction between classmates is mainly based on friendly relationships. One of the students points out: “the choice of the elements in group work is mainly based on the friendship between colleagues, available time and geographical proximity” (student of the computer engineering course). In fact, EI becomes even more relevant when we have to work in teams where we cannot choose their members or when our business duties force us to work and interact with people with very heterogeneous personalities. In these situations, high EI can be a determining factor for success in the job market. The study conducted in [45] emphasizes that people with higher EI present higher satisfaction from positive experiences and lower dissatisfaction from negative experiences.

The use of FLIGBY allows the assessment of students’ performance considering multiple dimensions. In the game, formative and summative elements are incorporated which, on the one hand, provide feedback on the decisions made by the player and, on the other hand, allow measuring and comparing the students’ performance. In fact, as mentioned in [22], immediate feedback is essential for reinforcing the success and correcting the errors. The assessment of EI skills in FLIGBY is performed through the use of performance measures. According to [20], this approach is preferable for use in self-evaluation reports, because it allows a more rigorous and complete external evaluation and avoids biased analysis.

The students’ performance in the “emotional intelligence” component was higher than the average of the 29 components considered in the MAP. Despite this, there was a greater dispersion of the results in the emotional intelligence component, with a wider range of results than those recorded in the average of the other 29 dimensions of MAP. This situation indicates that there were students with a very positive performance (maximum value obtained was 90), but also students who felt much more difficulties (minimum value equal to 55). This situation is pertinent in highlighting that the development of emotional skills is a field in which many students experience difficulties and have a lack of knowledge. Developing the ability to manage emotions and filter stressful stimuli is of utmost importance for students immediately in the school context, but also in the future in their professional context.

Our findings also indicate a significant correlation between students’ performance in the game and their score in “emotional intelligence” skill, in such a way that a good performance in this component originates, in 65.3% of the occurrences, a higher final score. The use of serious games in the development of emotional intelligence skills is an innovative area and, consequently, it is not possible to compare the findings with other studies in this field. Nevertheless, it is important to highlight the findings obtained in [46], which established a correlation between students’ EI and their academic performance. It is not possible to directly infer a relationship between the academic performance and a good performance in the serious game, but it is possible to explore the use of serious games in the development of emotional skills that, along the academic and professional path of students, may be important for their career. W also found that the performance of students in the game is independent of their course, gender, age, and number of years of professional experience. This situation is relevant because, although students with professional experience had a greater perception of the importance of EI, its final score in this component was not significantly different.

Despite the students’ positive results in the “emotional intelligence” component, they expressed difficulties in interacting with the characters of the game. In these situations, EI is even more decisive. Hence, several students have indicated that they have experienced evolution in the way they have been able to understand the opinions of the game characters and how the feedback received by the MR. FLIGBY allowed them to build healthier relationships in the teams and that manifested itself in the increased Flow of the team. One of the students points out: “Rebecca Saber was a great challenge due to her excessive concern for the company’s profits. Managing Rebecca’s relationship with Chris Strictland was complicated because they have very personalities and distinct visions for the future of the company” (student of the management course).

There was a consensus among the students that FLIGBY was a relevant instrument in the development of emotional skills. First of all, it allowed these students to understand how EI becomes important in a team. As CEO of Turul Winery, the students understood the importance of EI and how this component impacts the success of the company. In fact, this perception is confirmed in [14], which highlights the importance of business leaders being emotionally intelligent especially in a context where businesses depend heavily on interpersonal relationships. Nevertheless, the impact that EI will have on the students’ academic and professional future is difficult to quantify. However, the discussion generated in the semi-structured interview made it possible to identify that students with professional experience realize greater practical applicability in the short-term. One of the students highlighted: “Emotional intelligence allows me in the company that I work in the logistics area to motivate my employees in the face of fluctuations in deliveries, in order to keep them motivated and focused on periods of crisis in demand” (student of the management course). This observation is in-line with the work carried out in [47], which highlights that people with high EI skills are more easily integrated into a team and have better conditions to adapt to change.

## 5. Conclusions

In personal and professional interactions it becomes fundamental to recognize feelings or emotions, and know how to manage and apply them in different relationships and situations. By mastering these characteristics, the individual becomes more productive and contributes to the development into a successful professional. Despite the importance of EI, this is an issue that is little discussed in the context of higher education and many students have difficulties in evaluating themselves in this component. In this sense, the adoption of the FLIGBY serious game allowed us to evaluate the EI skills of students and helped them to develop these skills.

The findings allowed us to recognize that there is still some lack of knowledge regarding the significance of EI and its importance in the academic and professional context. The various challenges posed by FLIGBY throughout the 21 scenarios of the game and the different personalities of each character create different challenges to each student. The way each student used their EI skills was a determinant in the student’s final performance in the game, and the way they managed these skills was also a determinant to driving the team to the flow state.

This study offers both relevant theoretical and practical contributions. From the theoretical point of view, it was possible to assess the existence of a correlation between the player’s emotional skills and his/her performance in the game. It was also possible to conclude that variables such as the student’s course, gender, age, and years of professional experience were not determinants of the student’s performance in the EI component. Nevertheless, the data indicate that students with professional experience are those who more easily recognize the importance of emotional skills and realize greater practical applicability in the short-term, particularly within their professional organizations. From a practical point of view, this study is also pertinent in offering a process of evaluating students’ EI skills through a serious game.

With respect to future work, it becomes relevant to explore the impact of EI skills on the entrepreneurial motivation of students attending the discipline of entrepreneurship. Furthermore, it is also pertinent to explore the impact of EI on students’ academic path. At this level, it is expected that its impact can be potentially greater in subjects where greater control of emotions is required, for example, in disciplines where group work is relevant to students’ academic success. However, a study limitation is that the entrepreneurship discipline is given in the last year of the undergraduate courses. In this sense, it is difficult to assess its academic impact in their undergraduate courses. Therefore, an interesting approach is to explore the impact of emotional intelligence skills on the professional context and within postgraduate courses, as most of our students pursue postgraduate studies at our educational institution or at other partner institutions. Another limitation is the reduced number of students considered in this study because only students enrolled in the entrepreneurship discipline in the 2018/19 school year from two courses (i.e., management and computer engineering) were included. In this sense, efforts are already being made to ensure that in the next academic year this project will be continued with the involvement of students from the tourism course.

## Figures and Tables

**Table 1:** Sample descriptive analysis.

Dimension	Absolute Frequency	Relative Frequency
Course		
Management	18	0.3673
Computer Engineering	31	0.6327
Gender		
Male	36	0.7347
Female	13	0.2653
Age		
[18, 25]	24	0.4898
[25, 35]	14	0.2857
[35, 45]	8	0.1633
[45, 55]	3	0.0612
Years of professional experience		
0	21	0.4286
[1, 5]	10	0.2041
[5, 10]	12	0.2449
≥10	6	0.1224

**Table 2:** Characters in the FLIGBY game.

Name	Function
Bob Turul	Owner of the winery. Responsible for hiring the CEO (i.e., the player’s character).
Ellen McMason	Tasting room and hospitality manager. At the winery for 35+ years.
Joe Salleri	Public relations and events manager. He is Bob’s nephew.
Larry Turul	Assistant vineyard manager and assistant wine maker. He is Bob’s grandson.
Jen Goodwin	Executive assistant and back-office manager.
Rebecca Saber	Sales manager. Responsible for planning, directing and coordinating activities related to the sale of products.
Chris Strictland	Vineyard manager. Responsible for growing the grapes.
Alex Davenport	Chief winemaker. At the winery for over 20 years.

**Table 3:** Questions for semi-structured interview.

Dimension	Question
Background	Do you know the concept of EI?
	What is the relevance of EI on your academic and professional life?
Assessment	How do you look at your performance in “EI” variable?
	What situations in the game had the greatest impact on your performance?
Forecast	Do you consider that your EI skills have been improved during the game?
	What impact will the EI skills have on your career?

**Table 4:** Reliability analysis.

Cronbach’s Alpha	Cronbach’s Alpha Based on Standardized Items	Spearman-Brown Coefficient	Guttman Split-Half Coefficient
0.929	0.935	0.952	0.949

**Table 5:** Statistical analysis of sample data.

Statistic	Emotional Intelligence Dimension	All Dimensions
Mean	71.96	63.99
Median	73.00	63.75
Mode	77	60
Std. deviation	8.352	6.109
Skewness	−0.332	−0.082
Std. error of skewness	0.340	0.340
Kurtosis	−0.529	0.013
Std. error of kurtosis	0.668	0.668
Minimum	55	50.08
Maximum	90	77.90
Percentiles 25	66.00	60.53
Percentiles 50	73.00	63.75
Percentiles 75	78.00	68.45

**Table 6:** Linear regression.

R	R Square	Adjusted R Square	Std. Error of the Estimate
0.808	0.653	0.645	3.6388

**Table 7:** ANOVA analysis.

Dimension	Levene Statistic	Sig	Brown–Forsythe	Sig	F	Sig
Course	0.367	0.548	1.617	0.211	1.493	0.228
Gender	0.277	0.601	0.356	0.557	0.357	0.553
Age	1.354	0.269	0.673	0.583	0.803	0.499
Years of prof. exp.	0.522	0.670	1.132	0.356	1.320	0.280

**Table 8:** Results of the thematic analysis process.

Dimension	Final Themes
Background	Wrong concept of EI
	Confusing terminology
	Relevance in the academic context is not clear
	Relevance in professional life is clear
	Concrete examples of the importance of EI in professional life
Assessment	Performance was very positive
	Performance was increased through the several scenarios
	Heterogeneous identification with the characters in the game.
	Challenge level posed by each character was not homogeneous
Forecast	Consensus on development EI skills
	The impact may be high, although difficult to be quantified
	Students with professional experience see greater practical applicability in the short-term
